# Mechanisms of Phytoremediation by Resveratrol against Cadmium Toxicity

**DOI:** 10.3390/antiox13070782

**Published:** 2024-06-27

**Authors:** Barbara Mognetti, Francesco Franco, Chiara Castrignano, Patrizia Bovolin, Giovanni Nicolao Berta

**Affiliations:** 1 Department of Life Sciences and Systems Biology, University of Turin, Via Accademia Albertina 13, 10123 Turin, Italy; patrizia.bovolin@unito.it; 2 Department of Clinical and Biological Sciences, University of Turin, Regione Gonzole 10, 10043 Orbassano, Italy; francesco.franco@unito.it (F.F.); chiara.castrignano@edu.unito.it (C.C.); giovanni.berta@unito.it (G.N.B.)

**Keywords:** cadmium, resveratrol, antioxidant, phytoremediation, intoxication

## Abstract

Cadmium (Cd) toxicity poses a significant threat to human health and the environment due to its widespread occurrence and persistence. In recent years, considerable attention has been directed towards exploring natural compounds with potential protective effects against Cd-induced toxicity. Among these compounds, resveratrol (RV) has emerged as a promising candidate, demonstrating a range of beneficial effects attributed to its antioxidant and anti-inflammatory properties. This literature review systematically evaluates the protective role of RV against Cd toxicity, considering the various mechanisms of action involved. A comprehensive analysis of both in vitro and in vivo studies is conducted to provide a comprehensive understanding of RV efficacy in mitigating Cd-induced damage. Additionally, this review highlights the importance of phytoremediation strategies in addressing Cd contamination, emphasizing the potential of RV in enhancing the efficiency of such remediation techniques. Through the integration of diverse research findings, this review underscores the therapeutic potential of RV in combating Cd toxicity and underscores the need for further investigation to elucidate its precise mechanisms of action and optimize its application in environmental and clinical settings.

## 1. Introduction

Cadmium (Cd), a non-essential transition metal, poses health risks for both humans and animals. It can be released into the environment from natural events such as volcanic eruptions and forest fires or from anthropic sources, including agricultural and industrial activities. Exposure to Cd predominantly happens through consuming contaminated food and water, as well as through cigarette smoke and polluted air [1]. Chronic exposure to Cd among non-smokers primarily results from consuming contaminated food, while habitual tobacco smoking significantly increases Cd intake. A single cigarette contains between 0.5 and 2 μg of Cd, with 10–20% of the inhaled Cd being absorbed by the lungs. Smoking 20 cigarettes per day can result in the inhalation of approximately 0.2–4 μg of Cd. Dietary intake of Cd varies significantly based on diet composition and food quality, ranging from 10 μg to over 200 μg per day in industrialized countries, with 3–8% (and up to 30% in cases of dietary deficiencies) being absorbed in the intestines. Staple foods such as wheat, rice, and potatoes contribute about 40–50% of the ingested Cd [2]. WHO and FAO established the provisional tolerable monthly intake (PTMI) at 25 μg/kg b.w., corresponding to a daily intake of 56–62 μg for a 70 kg person [3].

Within biological systems, Cd predominantly occurs as the Cd^2+^ ion, which can exploit its Ca^2+^ mimetic properties to enter cells through various mechanisms, including ion channels and transporters. Specifically, due to the close similarity in ionic radii, Cd^2+^ can be absorbed through voltage- or receptor-operated Ca^2+^ channels [1]. One significant element of Cd toxicity involves the alteration of calcium metabolism, marked by decreased absorption of calcium in the intestines, heightened excretion of calcium in the urine, and negative calcium balance [4]. Cd is the closest analogue of Zn in the periodic table, so most of its biological activities are thought to be due to Zn replacement in transporters and structural and storage proteins [5,6]. In addition, Cd can share transport pathways with other divalent metals, such as Mn [7]. Once inside the cell, it can also interfere with the function of key biomolecules such as enzymes, receptors, and transcription factors. For instance, Cd binds to and activates the estrogen receptor α in the absence of estrogen, thus leading to increased breast cancer cell proliferation [8].

Dietary Cd is absorbed through transport systems involved in cation uptake. Complexed with peptides, small proteins, and phytochelatin, Cd is directly absorbed via transcytosis for direct absorption. Following absorption, dietary Cd enters the liver via the hepatic portal system, prompting the synthesis of metallothionein, a metal-binding protein rich in cysteine. Cd preferentially binds to metallothionein, forming a complex that mitigates the toxicity associated with free Cd ions [9]. Cadmium distribution within the bloodstream favors erythrocytes, with a minor fraction, below 10%, dispersed in the plasma, where it forms complexes with various biomolecules such as albumin, amino acids, and glutathione (GSH) or undergoes strong binding interactions with metallothionein [4]. Cd tends to accumulate in vital organs such as the liver and kidneys. Once accumulated, Cd can remain within these organs for more than 15 years, increasing the risk of renal and hepatic dysfunctions [10]. The placenta partially limits the distribution of Cd to the fetus; in fact, the concentration in cord blood is about half of that in maternal blood [11]. Cd excretion is primarily mediated by the kidneys, though at a very slow rate, resulting in a prolonged Cd half-life [10]. Other biological fluids such as saliva and tears can aid in the elimination of Cd: there is particular concern regarding the excretion of Cd through breast milk. Recent studies have shown that no barriers limit Cd passage from blood to breast milk [12]. The absorption of dietary Cd in women and children raises great concerns. Typically, these two groups of individuals have low body iron stores, which may increase Cd absorption and subsequent health risks [13]. Exposure to Cd during pediatric ages may contribute to the onset of neurodevelopmental disorders [12]. Exposure to environmental Cd has been linked to various malignancies, including breast, lung, prostate, nasopharynx, pancreas, and kidney cancer [14]. A correlation was also noted between markers of Cd exposure (found in blood and urine) and conditions such as coronary heart disease, stroke, peripheral artery disease, and atherogenic alterations in the lipid profile [2]. Additionally, some human pathologies seem to be linked to exposure to Cd, such as pulmonary edema, testicular damage, osteomalacia, and damage to the adrenals and hemopoietic system [1,2].

One of the primary mechanisms of Cd toxicity is the induction of oxidative stress, which occurs when there is an imbalance between reactive oxygen species (ROS) production and the cellular ability to detoxify ROS [15]. Excessive ROS can then damage lipids, proteins, and DNA, leading to cellular dysfunction and death. Additionally, Cd can also stimulate the production of ROS by activating NADPH oxidase and inhibiting enzymes such as superoxide dismutase and catalase [16]. Given the strong connection between oxidative stress and inflammation, numerous studies have investigated whether inflammatory processes can also mediate Cd toxicity. Cd can activate immune cells and induce the production of inflammatory cytokines such as interleukin-1β (IL-1β) and tumor necrosis factor-alpha (TNF-α). This inflammatory response can exacerbate Cd-induced oxidative stress and promote tissue damage [17]. Cd can trigger apoptosis through dual mechanisms: disrupting mitochondrial homeostasis and activating caspases [18,19]. Particularly, Cd^2+^ accumulation inside mitochondria impairs membrane potential, leading to mitochondrial swelling and subsequent apoptotic cell death. This kind of mitochondrial-dependent Cd-induced apoptosis has been observed in various cell types, including neurons [18]. Furthermore, in vitro studies conducted on kidney and liver cell lines revealed that Cd exposure directs cell fate toward caspase-dependent cell death. Mechanistically, these studies identified a significant reduction in the expression of key anti-apoptotic genes, such as Bcl-2, upon Cd exposure. Consequently, there is an upregulation in the expression of pro-apoptotic mediators, including cleaved-caspase3, cleaved-caspase-9, and cleaved-PARP, leading to the activation of apoptotic pathways [20]. Cd disrupts pathways involved in cell growth and survival, such as the phosphatidylinositol 3-kinase (PI3K)/Akt/mTOR pathway [21]. It can activate mTOR, a protein that plays a critical role in regulating cell growth and metabolism, and this activation has been implicated in Cd-induced cell death. Additionally, Cd can activate MAPKs, such as ERK and JNK, which are involved in regulating cell survival and apoptosis [21,22].

From what has been said, Cd toxicity is a complex process involving multiple mechanisms, whose understanding is critical for developing effective strategies to prevent or treat Cd-induced cellular damage. Natural compounds have gained significant attention in the last decades due to their various health benefits. Among them, resveratrol (3,5,4′-trihydroxy-trans-stilbene, RV) has been extensively studied due to its ability to counteract complex systemic diseases as well as toxicity derived from heavy metal exposure [23,24].

**Figure 1:**
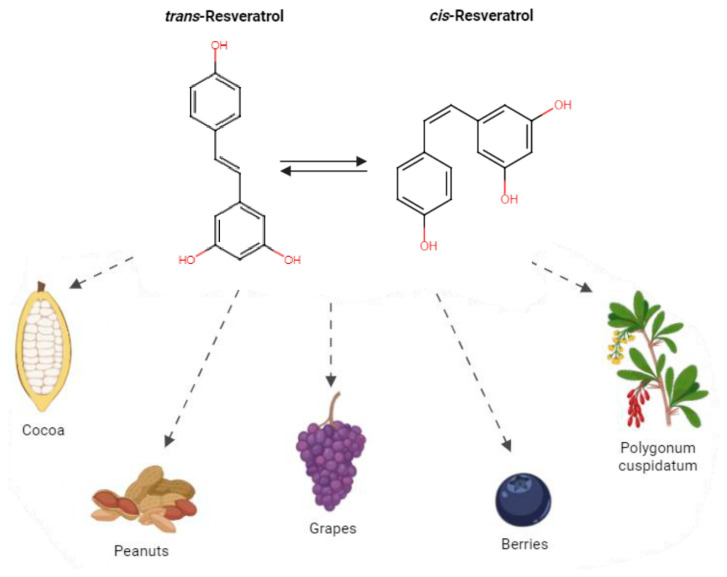
Structural formulas and sources of RV. This figure illustrates the structural formulas of cis-RV and trans-RV, two isomers of the polyphenolic compound RV. Additionally, it highlights various fruits which are known to be rich sources of RV. The cis and trans configurations refer to the spatial arrangement of the hydroxyl group (-OH) and hydrogen (H) attached to the carbon–carbon double bond in the RV molecule.

As a chemical entity, RV was first isolated in the 1940s from the roots of White hellebore [25] and later from *Polygonum cuspidatum*, a curative plant with a main role in Japanese and Chinese folk medicine [26]. In the following years, appreciable amounts of RV were identified in more than 70 other plant species, dietary supplements, and common edible fruits including pines, berries, legumes, and peanuts [27,28]. One of the richest RV sources is grape skin, and, consequently, it is present in wine, where Langcake and Pryce first evidenced it in 1976 [29]. In particular, RV is a non-flavonoid polyphenolic compound containing the stilbene skeleton. Stilbene-based compounds, widely represented in nature, have a C6–C2–C6 structure and are considered among the main non-flavonoids of dietary significance, which are the C6–C1 phenolic acids [30]. RV exists as the two structural isomers cis and trans, but the second is the most stable steric form, where the substituents are oriented in opposing directions, and also the most biologically active, maybe for its non-planar conformation (Figure 1). The trans form is more powerful in inhibiting the activity of enzymes such as DNA polymerases and cyclooxygenases (COXs), and it possesses more free radical scavenger capacity.

The wide range of possible RV-induced effects and its versatility lies in the multitude of targets it interferes with, such as cell-surface proteins, intracellular/nuclear receptors, membranes, transduction machinery signaling molecules, biogenesis enzymes, oxidative systems, DNA-repair mechanisms, and transcription factors [31,32,33]. RV is both a free radical scavenger (thanks to its hydroxyl groups which can scavenge free radicals produced in vivo) and a potent promoter of a variety of antioxidant enzyme activities; one of the highest affinity targets that has been identified is the enzyme quinone reductase 2 (QR2) [34]. In addition to its antioxidant properties, RV can also modulate various signaling pathways and enzymes and can inhibit the expression of pro-inflammatory cytokines and chemokines [32,33].

In 2022, the novel food trans-RV from a microbial source was authorized as a novel food ingredient under Regulation (EC) No 258/97 of the European Parliament and of the Council to be used in food supplements, in capsule or tablet form, for the adult population [35]. To date, the clinical application of RV has been examined in over 160 clinical trials, encompassing conditions such as diabetes mellitus, cancer, cardiovascular diseases, and neurodegenerative disorders [14,36,37]. 

Through this review, we have endeavored to delve into and understand the mechanisms of action through which RV appears to exert its protective effect against Cd toxicity. The findings of this study can contribute to the development of preventive and phytoremediation strategies using this molecule and offer valuable guidance for a natural approach to addressing Cd toxicity, ultimately improving human health and environmental well-being in the face of Cd exposure. Phytoremediation exploits the natural abilities of certain plants or plant-derived products to detoxify contaminants through various molecular mechanisms. This method is cost-effective and sustainable, making it an attractive alternative to conventional remediation techniques.

## 2. Materials and Methods

In February 2024, a comprehensive literature search was performed on three scientific databases, PubMed, Embase, and Google Scholar, using the keywords “resveratrol” and “cadmium”. Study identification and inclusion were carried out by B.M. and C.C., and any conflicts were resolved by a third author, G.N.B. The search generated 33 articles. Eight of these were excluded because they were not relevant (describing the development of new techniques, or Cd was used in the form of nanomaterials, or were not in English, or were review articles). Therefore, 25 articles were included, they are listed in Tables 1–5.

The experimental studies included in this review use Cd salts rather than elemental Cd, as it is insoluble in aqueous systems.

## 3. Central Nervous System

Among the different districts where Cd exerts its toxicity, the central nervous system has gained prominent attention within the scientific community. Cd toxicity is associated with a range of behavioral effects, including cognitive impairment, mood disorders, motor dysfunction, and hyperactivity, attributable to its neurotoxic impact. Cd toxicity in the central nervous system is principally due to oxidative stress, inflammation, and disruption of calcium homeostasis. Wang [38] suggests that Cd can disrupt neurotransmitter function, cause oxidative damage, and interact with other metals, while Arruebarrena [39] highlights its impact on zinc and calcium homeostasis, mitochondrial respiration, and neurotransmission. Hao [40] identifies the induction of ferroptosis and apoptosis by Cd, mediated by the miR-34a-5p/Sirt1 axis. Cd exposure has also been linked to a range of behavioral effects in both animals and humans: in rats, chronic exposure to Cd led to decreased locomotor activity and altered emotional reactivity [41]. Similarly, occupational exposure to Cd was associated with slower visuomotor functioning and increased complaints consistent with peripheral neuropathy [42].

In their studies, Shati administered CdCl_2_ only or CdCl_2_ and RV orally to adult male rats and checked their behavior. Functionally, it has been observed that the daily exposure of rats to 5 mg/kg CdCl_2_ for 45 consecutive days impairs retention and spatial memory [43] and decreases exploration time during training in the novel object recognition task (NORT) test, indicating impaired short and long-term recognition memory [44]; addition of RV 300 mg/kg body weight improved memory, latency during the passive avoidance test, and short and long-term recognition memory. When analyzing post-mortem brains of in vivo treated rats, it was found that the administration of CdCl_2_ in vivo [44] determined increased levels of p-Tau (Ser199 and Ser396) and p-GS3Kβ (Tyr216) with a decrease in p-GS3Kβ (Ser9), which suggests abnormal phosphorylation of Tau protein and activation of GSK3β; these two phenomena are suspected of contributing to the development of neurodegenerative disorders [45]. RV administration decreased the phosphorylation of Tau at Ser199 and Ser216 and p-GS3Kβ (Tyr216) while increasing p-GS3Kβ (Ser9) compared to control or CdCl_2_-treated rats. Co-administration of LY294002 (a selective pan-PI3K inhibitor) with RV + CdCl_2_ led to an increase in the levels of p-Tau (Ser199 and Ser396) and p-GS3Kβ (Tyr216) and a decrease in protein levels of p-GS3Kβ (Ser9) compared to CdCl2 + RV-treated rats. Rats exposed to CdCl_2_ showed decreased levels of SIRT1 activity, mRNA, and protein [43], decreased levels of GSH, and increased levels of GSH disulfide, malondialdehyde, and ROS, all oxidative stress markers. Those effects were reversed by co-administration of RV, thereby confirming its ability to decrease CdCl_2_-induced oxidative stress [43,44,46]. In addition, rats administered CdCl_2_ had increased levels of cleaved caspase-3 and caspase-12, which have been reverted by coadministration of RV [43,44]. The increase in the expression of activated caspase-3 after exposure to Cd and its dose-dependent reduction by RV treatment has also been confirmed by ex vivo experiments in primary neurons [46,47,48]. 

In the brain homogenates of rats, the levels of PI3K-p85α and Akt proteins were not significantly different between the experimental groups, but rats exposed to CdCl_2_ had decreased activation of these proteins compared to controls. Animals exposed to CdCl_2_ also had decreased levels of AMPKα1 and p-AMPKα1/2; on the other hand, the control and CdCl_2_-exposed rats co-administered with RV had increased levels of p-PI3K (Tyr697) and Akt (Ser473), as well as p-AMPKα1/2 and total AMPKα1, with higher activation ratios of these proteins compared to control rats or CdCl_2_-treated rats, respectively. The co-administration of LY294002 with RV to CdCl_2_-exposed rats significantly lowered protein levels of p-PI3K (Tyr697) and Akt (Ser473) to a similar level reported in rats exposed to CdCl_2_ but did not affect levels of AMPK or p-AMPK, confirming that AMPK is an upstream inducer of the PI3K/Akt signaling pathway [44]. Rats that were administered CdCl_2_ exhibited elevated levels of Bax, with a concomitant decrease in levels of Bcl-2 compared to control rats. Conversely, rats treated with RV demonstrated significantly increased levels of Bcl-2 and markedly reduced levels of both Bax and p-JNK in comparison to CdCl_2_-exposed rats. All the Cd-induced effects were reverted by the coadministration of RV [43,44]. The involvement of JNK in Cd toxicity has also been explored in an ex vivo model of primary cortical neurons isolated and cultured from fetal mice [47,48]. Based on previous studies showing that JNK and Erk1/2 participate in Cd-induced apoptosis in neuronal cells [49], Liu et al. [48] pre-treated primary neurons with RV (0–100 µM) for 1 h and then exposed them to Cd (10 and 20 µM) for 4 h. Western blot analysis showed that RV suppressed Cd-induced phosphorylation of JNK, c-Jun, Erk1/2, and p38 in a concentration-dependent manner. In addition, primary cultures of rat neurons exhibited morphological changes archetypal to apoptosis already 12 h after Cd exposure, with a significant reduction in cell viability. Pre-treatment with RV reduced the intensity of these changes and resulted in a significant improvement in cell viability percentage [43,46,47]. 

Analogous results have been obtained in a different animal model by Lv et al. [50]. This study found significant changes in the cerebrum structure and behavioral abnormalities in chickens exposed to Cd, which showed a depressed mental state, reluctance to exercise, and sensitivity to external stimuli. Histological analysis revealed that in Cd-treated chickens, the cerebral medulla was loosely enlarged, the number of granule cells increased, the cerebral cortex space was enlarged, and there was an increase in nuclear-disintegrated neurons. In contrast, in the Cd + RV group, the cerebral medulla was visibly loose, the nucleus was difficult to recognize, and the number of granule cells was reduced. Enzyme analysis indicated that Cd exposure suppressed antioxidant enzyme activity and increased oxidative stress markers, which were mitigated in the RV + Cd group. CYP450 and Cyt b5 levels were elevated in the Cd group but normalized with RV treatment. In Cd-treated chickens, the activities of aniline hydroxylase (AH) and NADPH-cytochrome reductase (NCR) were significantly increased. AH is an enzyme involved in the metabolism of aromatic amines, while NCR plays a key role in electron transfer during the oxidation of various substances by cytochrome P450 enzymes. The elevated activities of these enzymes indicate enhanced metabolic processing in response to Cd exposure. Additionally, gene expression analysis revealed that Cd exposure upregulated the downstream target genes of the nuclear receptors AHR (aryl hydrocarbon receptor), CAR (constitutive androstane receptor), and PXR (pregnane X receptor). These receptors are critical for detecting and responding to xenobiotic compounds, including pollutants like Cd. When activated, they induce the transcription of various cytochrome P450 (CYP) isoforms, which are enzymes involved in the metabolism and detoxification of foreign substances. This upregulation leads to increased transcription of CYP isoforms, indicating an enhanced metabolic response to Cd. In contrast, treatment with RV alongside Cd reduced the activities of AH and NCR, suggesting a normalization of metabolic enzyme activity. RV effectively countered the Cd-induced upregulation of AHR, CAR, and PXR target genes, thereby reversing the increased transcription of CYP isoforms.

Previous research demonstrated that Cd inhibits protein phosphatases 2A (PP2A) and 5 (PP5) [49], which act as negative regulators of MAPK signaling pathways. However, through Western blot analysis, Liu et al. have shown that pre-treatment of primary neurons with RV significantly reverses Cd-induced expression of demethylated- and phospho-PP2A, which are events associated with decreased PP2A activity. Therefore, they suggest that RV can prevent Cd from inhibiting PP2A and PP5 by rescuing their protein levels and attenuating demethylation and phosphorylation of PP2Ac. RV may therefore suppress Cd activation of JNK, Erk1/2, and p38 pathways by preventing Cd from inhibiting PP2A and PP5 [47,48]. The protective effect of RV acting through the activation of PP2A has been demonstrated also towards other neurotoxic molecules such as formaldehyde [51]. On the other hand, it has been demonstrated that celastrol, a plant-derived triterpene, exerts its neuroprotective action against Cd by preventing it from reducing the expression of PP5 [52].

Another neuronal mechanism disrupted by Cd via multiple pathways is intracellular calcium homeostasis [53,54]. It blocks voltage-gated calcium channels, impeding calcium influx crucial for neuronal signaling. It also prompts calcium release from intracellular stores like the endoplasmic reticulum and mitochondria, inducing cytosolic calcium overload, potentially leading to neuronal damage. Cd also interferes with calcium-binding proteins like calmodulin and parvalbumin, crucial for calcium signaling regulation, thus impairing neuronal function. Moreover, Cd exposure activates calcium-dependent signaling pathways associated with apoptosis, oxidative stress, and inflammation, exacerbating neuronal injury and neurodegeneration. Research has indicated that Cd disrupts the balance of intracellular free calcium ([Ca^2+^]i), resulting in apoptosis across various cell types, including primary murine neurons. Xu et al. [55] have shown that apoptosis triggered by Cd in primary murine neurons follows a pathway dependent on calcium signaling. When exposed to Cd, cerebral cortical neurons undergo morphological alterations that suggest apoptosis and eventual cell death. It also induces elevation of [Ca^2+^]i and inhibition of Na^+^/K^+^-ATPase and Ca^2+^/Mg^2+^-ATPase activities. In an in vitro study on primary rat cerebral cortical neurons [56], Cd-induced elevation of [Ca^2+^]i was suppressed by a blocker of the endoplasmic reticulum calcium channel, suggesting that endoplasmic reticulum-regulated Ca^2+^ is involved.

Lin et al. [46] evaluated the combined effect of RV and BAPTA-AM (a calcium chelator that can effectively bind and sequester intracellular calcium ions) on Cd-induced perturbation of [Ca^2+^]i homeostasis in primary rat cerebral cortical neurons. Incubating the cells with 10 mM BAPTA-AM 30′ before exposure to Cd prevented the elevation of [Ca^2+^]i. Moreover, incubation with 2-APB, an ER calcium channel blocker, suppressed the elevated levels of [Ca^2+^]i induced by Cd. RV alone caused a minor decrease in [Ca^2+^]i levels, but the combined exposure to BAPTA-AM and RV was more effective in suppressing the raised intracellular calcium levels. RV at 20 µM was found to be more effective in reducing the levels of [Ca^2+^]i [46]. These findings suggest that RV can mitigate the increase in intracellular calcium levels induced by Cd (Table 1). 

## 4. Reproductive System

Some epidemiological studies have been conducted to evaluate potential harmful effects of Cd on sex hormone levels, sperm parameters, and male infertility [57,58,59]. In a study conducted on a hundred Nigerian fertile and infertile men, the testes, along with the epididymis and seminal vesicles, were found to be among the most commonly Cd-targeted organs: indeed, azoospermic individuals were shown to have greater serum and seminal plasma Cd levels than oligospermic ones [60]. 

Based on these data, Mitra et al. [61] attempted to examine the harmful effects of two-week CdCl_2_ exposure (1.25 mg/kg b.w. and 2.5 mg/kg b.w.) on testicle anatomy and semen features in male Swiss albino mice. Cd exposure overwhelmed the tissue natural defenses, resulting in rapid damage in testicular histoarchitecture. Consequently, the researchers investigated the possibility that this would result in the upregulation of EGFR and its downstream signaling pathways and assessed RV ability to shield cells from CdCl_2_-induced damage. Indeed, the RV-pre-treated group exhibited recovered seminiferous tubule histoarchitecture, which is consistent with prior studies that found comparable effects in the testes of rats intoxicated with Cd [62,63]. In additional studies, long-term subcutaneous administration of Cd in mice has been linked to testicular cancers. When testis tissue treated with CdCl_2_ was compared to the control group, immunohistochemistry and Western blot analyses revealed a remarkably higher expression of EGFR, p-AKT, AKT1/2/3, NF-κβ, and COX-2 proteins. The results point to a direct correlation between testis morphological modifications and overexpression of these proteins. EGFR over-activation has been linked to an increase in PI3K/AKT signaling activity, a pathway that is frequently dysregulated in malignancies of the reproductive tract in both males and females [64]. Cd has been shown to increase EGFR protein expression cascade through various mechanisms. Ali et al. [65] found that nanomolar levels of CdCl_2_ chloride activate the Raf-MEK-ERK1/2 MAPKs signaling pathway via EGFR in human cancer cell lines. Lian et al. [66] further demonstrated that in human endothelial cells, Cd induces matrix metalloproteinase-9 (MMP-9) expression through ROS-dependent EGFR, NF-кB, and AP-1 pathways. Kundu et al. [67] reported that Cd upregulates EGFR and proinflammatory cytokines, leading to increased cell growth in a human lung adenocarcinoma cell line. Lastly, Chakraborty et al. [68] showed that chronic Cd exposure activates the Wnt pathway and upregulates markers of epithelial-to-mesenchymal transition in mouse kidneys, potentially contributing to renal fibrosis and cancer.

Overall, these results showed a marked rise in the proportion of sperm cells with aberrant morphology together with increased EGFR, p-AKT, AKT1/2/3, NF-κβ, and COX-2 expression in the CdCl_2_-only treated group. These proteins are likely crucial in hindering the process of spermatogenesis and causing histological disruption of testicular tissues. However, the precise significance of these correlations between protein overexpression, motile cell proportion and morphologically aberrant sperm cells is still under investigation. Notably, in RV-treated mice tissues, very little expression of EGFR and downstream proteins was detected, and there was no association between the percentage of sperm motility, protein overexpression, and morphologically aberrant cells. This indicates that RV pre-treatment can effectively repress Cd-induced cytotoxicity, as well as EGFR and downstream signaling protein upregulation. In order to determine whether a 16-week subchronic exposure to Cd can cause changes in testicular histoarchitecture, semen parameters, and expression of Akt cascade proteins culminating in the formation of germ cell neoplasia in situ, Mitra et al. conducted a follow-up investigation by using the Swiss albino mice model [69]. Their results revealed that the intracellular GSH/GSSG ratio is considerably decreased by subchronic exposure to low doses of CdCl_2_, which in turn alters the intracellular redox balance towards acute oxidative stress conditions. This finding aligns with prior research, as it has been observed that men exhibiting atypical semen parameters present a significantly reduced GSH/GSSG ratio [70]. Since it has been demonstrated that Cd engages with the PI3K/Akt cascade through ROS and non-ROS-mediated mechanisms [71,72], Akt and its downstream proteins NF-κB and Cox-2 were the main focus of the study. In fact, notable inverse correlations between the tissue GSH concentrations and the activated Akt cascade protein expressions were reported in mice that were exposed to two distinct CdCl_2_ concentrations (0.25 and 0.5 mg/kg) for 16 weeks. The authors demonstrated that extended treatment of 20 mg/kg of RV in conjunction with Cd can greatly enhance sperm motility, morphology, and density by promoting the synthesis of cellular GSH, which aligns with the outcomes of their prior investigation [61]. The advantageous in vivo benefits of RV extend beyond its ability to suppress NADPH oxidase-mediated ROS production, they also hinge on its strong chemo-preventive effect, with Akt being one of its main molecular targets [73,74]. Long-term alterations to cellular redox balance decreased the reproductive ability and led to the formation of testicular germ cell neoplasia in situ in mice exposed to Cd. However, intake of RV effectively countered the reproductive damage and carcinogenic effects induced by metal exposure in mice by restoring the oxidative balance in the teste tissue. It has become clear in recent years that the use of nutraceuticals in general may be an effective preventive and therapeutic strategy for mitigating the damage caused by Cd. To demonstrate the validity of this statement, Ferlazzo et al. [75] reported that bergamot juice, either by itself or in conjunction with RV and curcumin, could counteract the pro-inflammatory and oxidative effects of Cd as well as reduce its pro-apoptotic signals. After being exposed to Cd, mature male C57 BL/6J mice were given a flavonoid-rich antioxidant extract over a period of 14 days. Testes were then subjected to molecular, structural, and immunohistochemical investigations, which revealed that RV, curcumin, and bergamot juice at 40 mg/kg significantly increased Bcl-2, decreased p53, TNF-α, IL-1β, and BAX mRNA levels, and reversed tubular lesions and germinal cell death. Specifically, each parameter approached the control values when curcumin, RV, and bergamot juice were combined at dosages of 50/20/20 and 100/20/40 mg/kg. With conflicting information about the protective benefits provided by RV and curcumin [76,77] and no proof of bergamot juice’s potential contribution [78], the authors demonstrated that the three together alleviate the Cd-induced damage to the testes through a mechanism involving their anti-inflammatory and antiapoptotic properties. 

Eleawa et al. [79] observed that Cd administration increased lipid peroxidation and oxidative stress in the testes of treated rats; this has been linked to the observed testicular damage (decrease in sperm count and motility, with increased morphological abnormalities), along with a reduced hormonal level of testosterone, LH and FSH. The lower levels of gonadotropins could be explained by the fact that CdCl_2_ induces apoptosis in the anterior pituitary gland in a dose-dependent manner, consequently inhibiting steroid production. Authors were able to demonstrate that in both control and CdCl_2_-exposed male rats receiving daily RV, the two primary activities of the testes (the synthesis of steroid hormones and the production of spermatozoa), which are respectively regulated by gonadotropins and testosterone, were enhanced. Administration of RV had no negative effects since a daily dose of up to 300 mg/kg of RV for 28 days in adult rats was proven to be safe [80]. Pre- and post-treatment with RV restored testicular oxidative stress triggered by CdCl_2_, evidenced by a significant decrease in malondialdehyde levels and a notable increase in superoxide dismutase activity. Therefore, it appears that RV increases the production of sperm and androgens by lowering the levels of ROS and lipid peroxidation factors induced by CdCl_2_. Additionally, RV seems to induce genes related to mitochondrial biogenesis and oxidative phosphorylation, which increase the spermatozoa energetic metabolism and their motility and viability [81]. This work was the first to reveal that RV could increase, in both healthy and CdCl_2_-exposed rats, Bcl-2 mRNA expression levels and decrease p53 and Bax expression, indicating a potential mechanism of action in preventing damage to testicular tissue and germ cells. Their results support the work of Juan et al. [82], demonstrating that RV administration was able to enhance sperm production in rats by stimulating the hypothalamic–pituitary–gonadal axis without inducing adverse effects. A potential explanation for RV-induced hormone increase has been ascribed to its binding to the estrogen receptor as a mixed weak agonist/antagonist with no estrogenic properties [82]. 

In a similar manner to what occurred with the male reproductive system, the effects of Cd in vivo and in vitro have also been studied on the female reproductive system using animal models. Interference with the hypothalamic–pituitary–ovarian axis, decreased steroidogenesis, inhibition of follicle and oocyte development, impairment of ovulation and oocyte pick-up by the tubal epithelium along with retardation of embryo development and implantation, restricted fetal growth, and pregnancy complications are some of the detrimental consequences of Cd exposure [83]. The adverse effects of Cd exposure on oocytes meiotic competence during in vitro maturation, as well as on their capacity to successfully undergo in vitro fertilization and sustain preimplantation embryo development, have been documented by prior investigations employing mammalian models such as ovine, bovine, buffalo, and porcine [84,85,86,87]. The results of these investigations illustrate how the cytotoxic effect of Cd on in vitro maturation led to decreased rates of fertilization and an increase in polyspermy, as well as lower sperm binding capacity and an increase in the number of oocytes that were improperly fertilized. Furthermore, acute and chronic Cd exposure in female mice hindered oocyte meiotic development and lowered fertility by increasing ROS levels and apoptosis [88,89]. RV confirmed its antioxidant properties, preventing disruption of oocyte ROS homeostasis and a decline in their quality and improving in vitro maturation, fertilization, and embryonic development rates in various species [90,91,92,93,94]. The study conducted by Pira et al. [95] investigated the impact of RV addition to the maturation medium (1–2 μmol/L) on the in vitro maturation and fertilization of ovine oocytes while being subjected to Cd exposure. Given the reported benefits of RV, its effects on the quality of Cd-exposed in vitro matured oocytes were examined by evaluating chromatin configuration, intracellular ROS levels, mitochondrial activity and distribution, cytoskeleton morphology, and the mRNA expression of genes related to the cellular response to oxidative stress, such as SIRT1, SOD1, GPX1, GSR, and CAT. The reported data revealed that already 1 μmol/L of RV preserved oocytes against Cd-induced quality and function degeneration, thus avoiding improper maturation and fertilization. Of note, SIRT1 gene expression was considerably higher in oocytes treated with RV compared to controls. In human granulosa cells, SIRT1 activation by RV has been linked to anti-apoptotic effects, potentially contributing to the maintenance of oocytes [96]. RV has been found to improve the quality of pig oocytes through SIRT1 activation, enhancing ATP content and developmental ability [97]. These data might contribute to the comprehension of the underlying mechanisms of RV protection. 

From an epidemiological perspective, there is an increasing amount of information indicating that women have a larger body burden of Cd than men do, especially when it comes to pregnant women [98,99,100]. Preclinical studies performed in mice models showed that Cd exposure during gestation leads to placental accumulation, which ultimately causes fetal growth restriction and reduced fetal weight and placental weight [101]; in humans, exposure to Cd during pregnancy has been linked to lower birth weight in newborns [102]. 

Cd-induced fetal growth restriction and placental toxicity have been widely studied. Xu [103,104] found that maternal Cd exposure can lead to fetal growth restriction through the downregulation of glucose transporters and dysregulation of imprinted genes in the placenta. Levin [105] suggested that placental mechanisms, such as trophoblastic damage and altered blood flow, may contribute to Cd-induced fetal death. Zhu [106] identified a potential mechanism involving the triggering of PERK-regulated mitophagy in placental trophoblasts, leading to reduced progesterone levels and fetal growth restriction. Increased endoplasmic reticulum stress has been associated with impaired placental and fetal development [107]. Among the many mechanisms involved, it appears that the metal can ultimately activate Akt signaling in the placenta and cause apoptosis in human trophoblast cells and mouse placenta [108,109]. Another investigation [110] suggests that alterations in DNA methylation may mediate Cd toxicological effects on placental development. Moreover, placentas exposed to Cd had considerably higher DNA methyltransferase (DNMT) 3B and 3L expression levels, which are essential for DNA methylation [103]. This same enzyme can be downregulated by RV [111] as well as PI3K/Akt [32]. Jiang et al. [112] reported enhanced DNMT enzyme activity and genomic DNA methylation levels in lung fibroblast cells after Cd exposure, as well as upregulated DNTMT3 mRNA levels. RV has previously been demonstrated to reduce DNMT3 expression, and analogs have been designed as DNMT inhibitors [111]. Wang et al. [113] confirmed that RV was in fact able to suppress Cd-triggered increased DNMT activity and hindered the Cd-induced expression of DNMT3. Interestingly, they noticed RV recovered the expression of SIRT1, a histone deacetylase that modulates DNMTs activity. Results reported protective properties of RV at a concentration of only 20 μM, efficiently suppressing DNMT3B expression and activity in JEG-3 cells. However, this work only suggests the possible use of RES as a treatment for Cd toxicity. Although RV use during pregnancy has been proven to improve both maternal and placental phenotypes, it is worth mentioning that it alters fetal pancreas development [114]. 

Based on this evidence, Wang et al. [113] investigated the impact of RV on Cd-induced placental toxicity and examined the rationale underlying its protective effect, confirming that RV was inhibiting the activation of the Akt pathway [108]. RV anti-inflammatory action in the placenta is likely due to its inhibitory effect on Akt activation, as the pathway is involved in Cd upregulation of TNF-α, IL-8, and IL-6 mRNAs in JEG-3 cells (Table 2).

## 5. Kidneys and Liver

Among the various organs threatened by Cd toxicity, kidneys are considered the most susceptible mainly because of the absence of a specific mechanism for Cd elimination, particularly within renal glomeruli and their associated proximal tubules [115]. Consequently, after chronic exposure to Cd, approximately 50% of its total body stores accumulate in the kidneys, which can be significantly impacted by Cd exposure, with potentially life-threatening effects [116,117,118]. 

Multiple models have been employed to understand the nephrotoxic effects of Cd. As reported by Cirmi and colleagues, mice in vivo manifest distinct signs of nephrotoxicity after exposure to CdCl_2_ [17]. Specifically, common hematological markers of renal dysfunction such as urea, nitrogen, and creatinine increased after intraperitoneal 2 mg/kg Cd administration. By administering compounds of natural origin, which include RV, curcumin, and bergamot juice, either alone or in combination, they limited Cd-induced nephrotoxicity as demonstrated by an improvement in blood urea, nitrogen, and creatinine levels. The effects of those nutraceuticals mitigated Cd-induced overexpression of important regulators of both apoptosis and oxidative stress. Specifically, the administration of RV in mice exposed to Cd hampered the expression of pro-apoptotic genes such as tp53 and Bax, favoring at the same time an increase in the expression of the anti-apoptotic gene Bcl-2. RV also ameliorated the Cd-induced oxidative stress favoring an increase in the expression of two important antioxidant proteins: GSH and GPx. 

Analogous results have been obtained in a rat model of Cd-induced renal damage [119], where four weeks of Cd exposure result in a marked reduction in kidney weight, significant increase in markers of kidney dysfunction, including BUN, SCR, and NAG, and severe morphological changes such as glomerulus shrinkage, tubule dilation, and collagen deposition. RV administered at 20 mg/kg/day mitigates kidney dysfunction by lowering BUN, SCR, and NAG levels. Moreover, Cd exposure promotes epithelial–mesenchymal transition (EMT) in the kidneys, indicated by heightened levels of TGF-β1, twist, and fibronectin, leading to fibrosis. Furthermore, RV attenuates the expression of EMT markers, thereby preventing renal fibrosis.

Similarly to what was found in other organs and districts, Cd also triggers extensive oxidative stress in the kidney, characterized by increased levels of MDA, T-CO, and GSSG, along with reduced levels of T-SH and GSH. The activities of critical antioxidant enzymes like SOD, CAT, GR, and GPx are significantly diminished. This oxidative stress is further exacerbated by elevated inflammatory markers, including COX-2, iNOS, PGE2, and NO. RV antioxidant properties are evident as they decrease oxidative stress markers and increase antioxidant enzyme activity. It also reduces inflammation by lowering COX-2, iNOS, PGE2, and NO levels [119].

These data were corroborated by Fu et al. [120], who administered Cd and RV in vivo in a murine model. In line with previous research, they noted a substantial rise in blood markers of renal damage (blood urea nitrogen and serum creatinine) following Cd administration in mice. RV effectively thwarted the increase in these markers, mitigating Cd-induced renal damage. To understand the phenomena observed in vivo and in light of recent findings that suggest malfunctioning mitochondria play a central role in renal damage [121], Fu and colleagues studied in vitro the effects of Cd on oxidative stress and mitochondrial function on murine renal tubular epithelial cells (TCMK-1) [120]. The results suggest that Cd exposure induces widespread mitochondrial dysfunction in TCMK-1 cells, characterized by a significant increase in the production of mitochondrial-derived reactive oxygen species (mROS). Additionally, Cd exposure leads to a reduction in the expression and activity of Sirt3, resulting in the suppression of its downstream target proteins, including FoxO3a, PGC-1α, and SOD2. RV has been shown to restore the expression of Sirt3 in Cd-exposed TCMK-1 cells facilitating the expression of FoxO3a, PGC-1α, and SOD2, thereby enabling mROS detoxification. The authors mechanistically linked Cd-induced renal damage in mice to apoptosis, triggered by hyper-phosphorylation of ERK1/2. Administration of RV in Cd-exposed mice significantly reduced ERK1/2 phosphorylation in the kidneys, thereby alleviating apoptotic cell death.

Zang and colleagues conducted an in vivo study to investigate the impact of Cd exposure and the subsequent oxidative stress on the kidneys of white chickens [122]. The birds were exposed to Cd for 90 days, after which their kidneys underwent histopathological analysis. The exposure to Cd led to severe structural damage in the kidneys, including glomerular atrophy, nuclear lysis, nuclear fragmentation, and pyknosis. Additionally, a significant decrease in the expression of antioxidant enzymes such as T-SOD, Cu-Zn SOD, GSH-Px, GST, and CAT was observed. RV dramatically alleviated Cd-induced histopathological renal lesions. Moreover, the administration of RV to Cd-exposed animals resulted in the restoration of antioxidant enzyme expression levels that were reduced by Cd exposure. Since a correlation between oxidative stress and the activation of inflammation has been demonstrated [123,124], Chou et al. investigated whether Cd exposure might exacerbate inflammation in the kidneys [125]. Using a well-established in vitro model of human proximal tubular epithelial cells (HK-2), they observed the activation of the NLRP3 inflammasome following Cd exposure. The NLRP3 inflammasome is responsible for the activation and secretion of potent inflammatory mediators such as IL-1β and IL-18. Thus, the activation of the NLRP3 inflammasome triggered by Cd exposure initiates a significant inflammatory response in NK-2 cells that ultimately leads to pyroptotic cell death [126]. Exposure to Cd results in the downregulation of SIRT1 expression and activity, which leads to the accumulation of acetylated XBP-1 and activation of the IRE-1α/XBP-1s pathway. This phenomenon stimulates the NLRP3 inflammasome, promoting inflammation and triggering pyroptosis. The administration of RV, a SIRT1 activator [127,128,129], mitigates the pro-pyroptotic effects of Cd. 

In summary, Cd exposure causes significant renal damage through oxidative stress, inflammation, and fibrosis and, as in other districts, RV offers a robust protective effect against Cd-induced renal injury, reducing its adverse outcomes.

The liver is another target of Cd toxicity. Several studies have reported that Cd exposure can induce significant adverse events, including oxidative stress, mitochondrial dysfunction, and inflammation [130]. Aiming at identifying a possible protective effect of RV toward Cd-induced hepatotoxicity, Rafati and colleagues administered to adult mice Cd alone and in combination with RV [131]. The results of the study revealed that exposure to Cd caused significant hepatocellular degeneration characterized by an increase in the volume and number of hepatocytes, as confirmed by histological analysis. Additionally, there was a notable decrease in the volume of sinusoids and central veins in the livers of Cd-exposed mice. Stereological analysis further revealed a threefold increase in fibrous tissue. However, co-administration of RV mitigated the structural changes induced by Cd exposure, attenuating hepatocellular degeneration and fibrotic transformation, thus preserving the normal architecture of liver tissue. 

The molecular mechanisms of RV protection toward Cd hepatotoxicity have been further explored by Eybl et al. [132], who demonstrated that the administration of CdCl_2_ led to a significant increase in malondialdehyde concentration in the liver, indicating increased lipid peroxidation and confirming the oxidative stress. However, pre-treatment with RV completely prevented this increase. Cd exposure reduced hepatic GSH and GSH peroxidase (GPx) activity to about 60% of control levels and catalase activity to 68% of control levels. Pre-treatment with RV nearly completely restored GSH and GPx to normal levels and ameliorated the reduction in catalase activity, restoring it to 89% of control levels. Notably, pre-treatment with RV did not influence Cd accumulation in the liver, kidneys, or brain, suggesting that its protective effects stem from bolstering antioxidant defenses rather than modifying Cd distribution.

Al-Baqami et al. [133] obtained consistent results studying the toxic activity of Cd on the livers of Albino rats. Similarly to what is observed in the murine model, Cd exposure in Albino rats triggers liver injuries, evident from the increase in liver-damage related proteins and histological damages. Interestingly, when Cd was administered together with RV, the authors observed an improvement in both hematological and histological markers of liver damage (Table 3).

## 6. Thyroid

Cd has been classified as an endocrine-disrupting chemical, and the thyroid has been proven to be one of the targets of its detrimental effects [134,135,136]. Despite conflicting and limited evidence in the literature, it appears that Cd not only can cause hypothyroidism but also affects follicular C cells [137,138]. Adult rats given prolonged Cd treatment showed C cell proliferation, which seems consistent with the observations of C cell diffuse hyperplasia, hypertrophy, and hypergranulation [139]. Animals who were given an acute dose of Cd experienced a sharp reduction in calcitonin expression in C cells [140]. Based on this evidence, Benvenga et al. sought to demonstrate if RV protects the C cells against Cd exposure [141,142]. They demonstrated that RV treatment augmented the mean follicle diameter and counteracted the epithelium height increase induced by exposure to Cd. It also reduced hyperplastic and hypertrophic changes in C cells, decreased cytoplasmic density, and increased TUNEL-positive cells, indicating that normal apoptotic activity was restored (Table 4). 

## 7. Multiorgan Impact

In addition to the aforementioned systems and organs, Cd also exerts notable toxicity on the skeletal system. Cd can increase bone resorption, affect the activity of osteoclasts and calcium absorption, and impair kidney function, which favors the development of osteoporosis [143]. Its accumulation in bones [144,145] influences bone maturity and differentiation [146,147], thereby potentially reducing the survival rate of preosteoblasts and ALP activity. The injury to osteoblasts caused by Cd [148], coupled with oxidative stress, leads to DNA damage, mitochondrial dysfunction, and endoplasmic reticulum stress, ultimately culminating in apoptosis [149]. The effect of Cd on the osteoblastic maturation and proliferation of MC3T3-E1 cells was studied [150], and the possible relationship between Cd-induced bone toxicity and the MAPK pathway was investigated. Confirming precedent studies [151,152], Mei et al. [150] showed that exposure to CdCl_2_ significantly reduced cell viability and ALP activity and inhibited the osteoblastic differentiation of MC3T3-E1 cells. Furthermore, protein levels of p-P38, p-ERK1/2, and p-JNK were markedly higher after CdCl2 treatment, suggesting that the inhibition of cell differentiation induced by Cd is associated with the stimulation of the P38, JNK, and ERK1/2 signaling pathways. CdCl_2_ treatment increased the number of cells in early and late apoptosis and necrosis, but pre-treatment with RV significantly reduced the rate of apoptosis and prevented CdCl_2_-induced changes in cell morphology. Immunofluorescence staining and Western blotting showed that the RV + Cd group had decreased ERK1/2 and JNK compared to the Cd group. RV treatment also increased cell viability and ALP synthesis and rescued the reduction in ALP, RUNX2, COL1, and BMP-2 mRNA and protein levels induced by exposure to CdCl_2_. These results suggest that RV might reverse the CdCl_2_-induced inhibition of bone formation by inhibiting ERK1/2 and JNK signaling.

Among the best-described pathologies related to Cd exposure are cardiovascular diseases. To investigate Cd-induced myocardial hypertrophy and cardiomyocyte injury, Sasikumar et al. [153] established an in vitro model using the rat cardiomyoblast cell line H9c2, which enabled them to study the toxic effects of Cd and the protective effects of RV. They demonstrated that RV significantly increased the live cell population in Cd-exposed cells (*p* < 0.001), improving cell viability and antagonizing Cd-induced cardiotoxicity. In this system, the ability of Cd to induce ROS formation and the antioxidant action of RV were also confirmed, further demonstrating RV potential to counteract oxidative stress. Cd exposure led to a significant reduction in mitochondrial membrane potential (MMP), but RV restored MMP levels. Morphological analysis revealed that Cd-exposed cells exhibited structural alterations characteristic of apoptosis, which were reduced by exposure to RV. Additionally, Cd exposure increased the cell surface area and upregulated myocardial hypertrophy markers ANP, BNP, and βMHC. RV treatment reversed these hypertrophic gene expressions, indicating a reduction in myocardial hypertrophy. Finally, the expression of Nrf2-regulated genes such as HO-1, NQO1, SOD, and CAT was evaluated. While Cd exposure resulted in moderate Nrf2 expression and downregulated CAT, RV treatment significantly increased the expression of these genes, enhancing the protective response against Cd-induced myocardial hypertrophy. Overall, RV significantly ameliorated Cd-induced oxidative damage and pathological changes in cardiomyocytes.

In addition to the organ-specific toxicity considered in the previous paragraphs, Cd exerts generic toxicity in the form of carcinogenesis, with studies demonstrating its ability to induce tumors in both local and systemic sites including the lung, breast, prostate, testes, and kidney [154,155,156]. The exact molecular mechanisms of Cd carcinogenesis have been thoroughly described [157] and include stimulation of epithelial–mesenchymal transition (EMT), migration, and transformation [158]. Qian et al. [159] tested the effect of different concentrations of Cd on the migration and invasion of colorectal cancer (CRC) cells. Results showed that the migratory and invasive ability of CRC cells significantly increased with exposure to Cd. However, co-treatment with RV and Cd reduced the number of migrated and invaded cells. Higher RV concentrations increased the degree of change in migratory and invasive ability. EMT plays an important role and is currently one of the hotspots in the research on the mechanism of malignant tumor development; therefore, Qian et al. [159] also analyzed the effect of RV on the regulation of EMT-related markers in Cd-exposed CRC cells. Compared to the control group, Cd-exposed cells had up-regulated levels of Zinc finger E-box binding homeobox 1 (ZEB1), vimentin, and E-cadherin and down-regulated levels of N-cadherin. However, the high-concentration RV groups (100 and 200 μM) showed decreased levels of ZEB1, vimentin, and N-cadherin and increased levels of E-cadherin, in contrast to the Cd-exposed CRC cells. Moreover, they found that RV suppressed the m6A modification of ZEB1 and reduced the Cd-induced up-regulation of ZEB1 expression at the mRNA level. According to this study, RV could reverse the Cd-promoted migration, invasion, and EMT via m^6^A modification, which may be the mechanism of RV in reducing the occurrence and development of CRC (Table 5).

**Figure 2:**
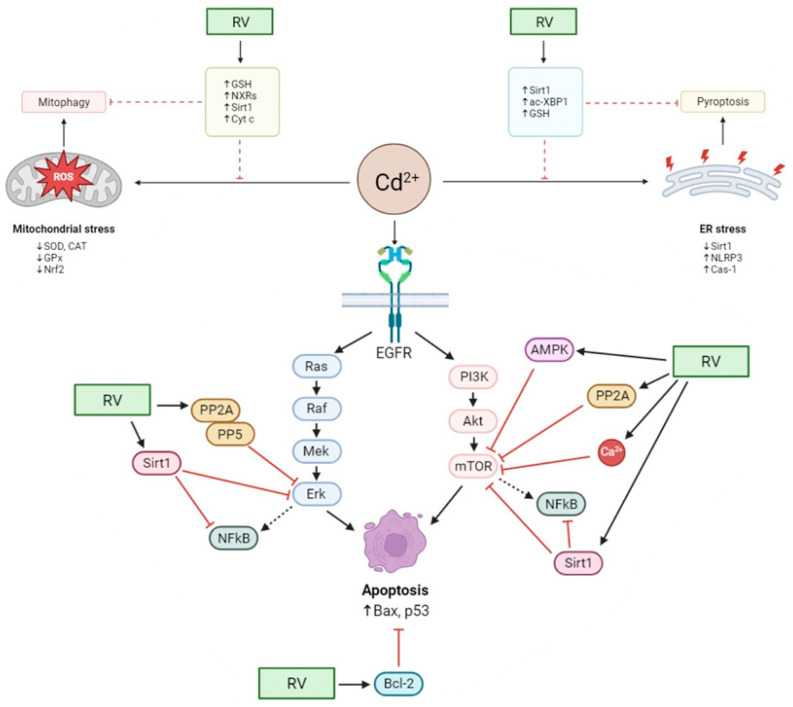
Molecular mechanisms of RV in reducing Cd toxicity at the cellular level. Cd can disrupt the oxidative/antioxidative status of cells, indirectly resulting in oxidative stress via weakening the antioxidative barrier by inducing ROS formation and increasing electron leakage from the mitochondrial respiratory chain, ultimately leading to mitophagy. The highly stressed cell state is also reflected in the ER, where inflammasome formation and activation occur due to the altered metabolic/enzymatic environment, eventually leading to pyroptosis. RV antioxidant action can revert stressful circumstances in both organelles by reducing ROS generation and counterbalancing the inflamed environment by activating antioxidant pathways and enhancing genes relevant to the cellular stress response via epigenetic modifications. At the molecular level, Cd action also activates various pathways, initiating defense responses and leading to apoptosis by over-activating EGFR and acting on multiple points of its signaling cascades. RV beneficial effects on the cell rely on activating specific effectors that exert their inhibitory potential directly on the most critical components of signaling cascades, effectively restoring phenotype by lowering the detrimental effects of their aberrant activation.

Figure 2 summarizes all the mechanisms by which RV counteracts Cd toxicity, as described in the reviewed literature. 

## 8. Conclusions

Cd is a well-known environmental and occupational pollutant that poses significant public health challenges worldwide [160]. Chronic exposure to Cd is of particular concern due to its ability to accumulate in the body and cause long-term health effects, such as kidney damage, bone demineralization, endocrine disruption in the reproductive axis, and an increased risk of cancer [161,162]. Cd induces oxidative stress, which plays a crucial role in its genotoxicity and carcinogenicity. Although Cd does not directly generate free radicals, it disrupts cellular oxidative balance by impairing antioxidant defenses and displacing essential metals like copper and iron, thereby promoting the formation of ROS. These disturbances lead to oxidative damage to cellular macromolecules, including lipids, proteins, and DNA, as well as to cellular structures and organelles, contributing to cellular apoptosis and necrosis [163].

Cd exposure occurs through environmental and occupational sources, with epidemiological data indicating widespread exposure and associated health risks. According to Chen et al. [15], about 10% of the global population is at risk of chronic low Cd exposure. Higher blood Cd concentrations are significantly associated with increased risks of all-cause mortality (HR 1.473), cardiovascular disease mortality (HR 1.445), and cancer mortality (HR 1.496) [164]. Machine learning feature selection ranked Cd as the second most important factor for all-cause and cancer mortality, following age. The association between Cd levels and all-cause mortality was significant in both ever-smokers and never-smokers. Smoking was estimated to mediate 32% of the effect of Cd on mortality, while 68% was attributed directly to Cd itself. A population-based cohort study investigated the association between Cd exposure and mortality [165], and hazard ratios for all-cause mortality were observed. Meta-regression revealed a 17% increase in relative risk per doubling of urinary Cd concentration. An interesting overview of Cd concentration in the blood and urine of the general population in various countries all over the world has been published by Mezynska and Brzóska [2].

Since Cd intoxication is predominantly chronic, it is ideal to introduce substances that can counteract its effects on a daily basis. The most effective approach to mitigating Cd poisoning would be eliminating human-produced sources of Cd, which requires strict regulations and sustainable practices in agricultural and industrial sectors, which is not always feasible. Therefore, it is useful to identify strategies to prevent or limit Cd toxic effects. A viable strategy could be phytoremediation, by introducing foods containing substances capable of limiting or repairing the progressive damage caused by Cd. Among the molecules potentially indicated for this purpose, in this review, we have considered RV for its already widely demonstrated antioxidant properties [24]. RV is a polyphenol found in several foods, including grapes, berries, peanuts, and red wine, with red wine and grape skins being particularly notable for their high RV content [166,167], thus making them ideal candidates for dietary inclusion to combat oxidative stress and inflammation associated with Cd toxicity. According to the articles we included in this review, RV exhibits significant potential in mitigating Cd-induced oxidative damage due to its potent antioxidant properties. It not only enhances the overall antioxidative capacity of cells but also specifically counteracts the oxidative mechanisms triggered by Cd exposure [24,168]. Our review highlights RV multifaceted antioxidative mechanisms, including scavenging free radicals, upregulating endogenous antioxidant defenses, and modulating signaling pathways involved in oxidative stress responses.

Unfortunately, the data we have, although promising and convincing, are only preclinical, and we do not have studies that demonstrate the effectiveness of RV in preventing or repairing the damage caused by Cd exposure in humans. Current evidence is insufficient to conclusively determine whether dietary or supplemental RV can provide significant protection or therapeutic benefits in cases of Cd poisoning. This uncertainty is partly due to the limited number of clinical studies on RV efficacy in such contexts.

The clinical application of RV faces challenges due to its pharmacokinetic properties, such as low bioavailability and rapid metabolism, which limit its therapeutic potential [24,169]. Consequently, ongoing research aims to enhance the bioavailability and stability of RV through various formulations and delivery systems to improve its clinical efficacy [170,171,172]. Advancements in biotechnology have paved the way for the development of promising nano-based delivery systems for natural compounds like RV. These systems include polymeric, lipidic, and inorganic nanoparticles, liposomes, nanotubes, and cyclodextrins, which can improve RV pharmacokinetic properties. Such nano-based delivery systems can significantly increase the bioavailability of RV, as demonstrated by several studies witnessing a burgeoning interest in the potential therapeutic properties of RV, particularly in light of the enhanced bioavailability achieved through innovative nanotechnology-based delivery systems.

In conclusion, while RV shows promising potential in reversing Cd-induced oxidative damage, clinical studies are urgently needed to validate its effectiveness and safety in humans. Improving the bioavailability of RV through advanced delivery methods will be pivotal in translating preclinical findings into viable clinical interventions.

## Figures and Tables

**Table 1:** Comprehensive summary of experimental designs employed in the articles reviewed in the central nervous system section.

Article	Experimental Target System	Dosage		Exposure Time		Administration Route		Ameliorative Effects of Resveratrol
		Cd	RV	Cd	RV	Cd	RV	
Shati et al. (2019) [44] *	AMPK/PI3K/Akt pathway, PP2A, and GSK3β	5 mg/kg	300 mg/kg	45 days	45 days	Oral	Oral	↑ cell survival; SIRT1 activity, mRNA, and protein; p-AMPK (Thr172) and p-Akt (Ser473); GSH and Bcl-2 ↓ cell apoptosis and ROS content; GSSG and MDA; Bax, cleaved caspase-3, cleaved caspase-12 and p-JNK; GAAD 153, GRP78, and ATF-6
Shati et al. (2019) [43] *	SIRT1/AMPK/Akt, ROS, Bcl-2, ER stress, GAAD 153	5 mg/kg	300 mg/kg	45 days	45 days	Oral	Oral	↑ learning and memory formation; GSH; Bcl-2; Ach and ChAT activity; p-PI3K and p-Akt; AMPKα1 and p-AMPKα1/2; activation ratio of p-PP2A ↓ ROS, MDA, and GSSG; Bax and cleaved caspase-3; AchE activity
Lin et al. (2015) [46] ^§^	MAPK/mTOR	10–20 μM	5–10-20 μM	12 h	12 h	NA	NA	↑ cell viability, PTEN ↓ apoptosis; [Ca^2+^]I; ROS, pAkt, S6K, and 4E-BP1; phosphorylation of JNK, ERK1/2, c-Jun, and p38 MAPK
Liu et al. (2015) [47] ^§^	Erk1/2, JNK, PP2A/5	10–20 μM	0–400 μM	24 h	24 h	NA	NA	↑ cell viability, PTEN, PP2A, PP5 ↓ nuclear fragmentation and condensation; TUNEL-positive cells, cleaved caspase-3; phosphorylation of JNK, c-Jun, p38, and Erk1/2
Liu et al. (2022) [48] ^§^	mTOR and neuronal apoptosis	10–20 μM	100 μM	4 h	1 h	NA	NA	↓ apoptotic cells; ROS; p-Akt; p-mTOR; p-S6K1; p-S6; p-4E-BP1; caspase-3 cleavage; caspases 3/7 activation; p-Erk1/2; p-JNK; TUNEL-positive cells
Lv et al. (2023) [50] *	antioxidant enzymes, CYP450 activity, and NXR-AHR-CYP1 pathway	140 mg/kg	400 mg/kg	90 days	90 days	Oral	Oral	↑ antioxidant enzymes (Cu-Zn SOD and T-SOD); Cyt b5; AH and NCR activities; AHR, CYP1A1/2, CAR/PXR, and CYP2&3 mRNA and protein levels ↓ ERND and APND activities

*: in vivo; ^§^: in vitro; NA: not applicable; ↑: increase; ↓: decrease/inhibition.

**Table 2:** Comprehensive summary of experimental designs employed in the articles reviewed in the reproductive system section.

Article	Experimental Target System	Dosage		Exposure Time		Administration Route		Ameliorative Effects of Resveratrol
		Cd	RV	Cd	RV	Cd	RV	
Mitra et al. (2016) [61] *	EGFR, Akt, NF-κB	1.25–2.5 mg/kg	10 mg/kg	3 times/wk	14 days	IP	Oral	↑ body weight; motile cells, live sperm cells ↓ morphologically abnormal cells, necrosis, germ cell derangement, and epithelium vacuolization; EGFR, p-AKT, AKT1/2/3, NF-κβ (p50), COX-2
Ferlazzo et al. (2021) [75] *	ROS, inflammation, apoptosis	2 mg/kg	20 mg/kg	Daily	14 days	IP	Oral	↑ body weight; testes weight; Bcl-2 ↓ edema in extratubular compartment; TUNEL-positive cells in seminiferous tubules; expression of TNF-α and IL-1β mRNA; Bax
Mitra et al. (2022) [69] *	AKT and GCNIS markers	0.25–0.5 mg/kg	20 mg/kg	2 times/wk	16 weeks	IP	Oral	↑ body weight; testes weight, sperm motility, viability, and morphology; GSH ↓ Akt, p-Akt, NF-kB, Cox-2; MDA, GSH ratio; expression of GCNIS markers
Eleawa et al. (2014) [79] *	Bcl-2, p53, Bax	1 mg/kg	20 mg/kg	Single	15 days	IP	Oral	↑ sperm parameters (count, motility, daily production); hormonal levels (FSH, LH, testosterone); diameter of seminiferous tubules; increased SOD activity, Bcl-2 mRNA expression ↓ testicular degeneration and necrosis, p53 and Bax mRNA expression
Piras et al. (2022) [95] ^§^	Maturation process and fertilization	2 μM	1–2 μM	-	-	NA	NA	↑ oocyte maturation and fertilization rates; SIRT1, SOD1, GPX1; ↓ polyspermic fertilization; mitochondrial activity; ROS
Wang et al. (2021) [113] *	DNMT3 and PI3K/Akt	4.5 mg/kg	300 mg/kg	Single	18 days	IP	Oral	↑ fetal weight and growth; placental diameter; estradiol secretion ↓ apoptosis and inflammatory response in placental cells; DNMT activity and expression; Akt signaling pathway; endoplasmic reticulum stress

*: in vivo; ^§^: in vitro; NA: not applicable; IP: intraperitoneal; GCNIS: germ cell neoplasia in situ; ↑: increase; ↓: decrease/inhibition.

**Table 3:** Comprehensive summary of experimental designs employed in the articles reviewed in the kidney and liver section.

Article	Experimental Target System	Dosage		Exposure Time		Administration Route		Ameliorative Effects of Resveratrol
		Cd	RV	Cd	RV	Cd	RV	
Cirmi et al. (2021) [17] *	ROS, inflammation, apoptosis	2 mg/kg	20 mg/kg	Daily	14 days	IP	Oral	↑ GSH and GPx levels; Bcl2 expression; SIRT1 ↓ apoptosis, oxidative stress, and inflammation; urea nitrogen and creatinine levels; tp53, Bax, DNMT3B, DNMT3L, Nos2, and Il1b gene expression; Akt signaling pathway
Hu et al. (2017) [119] *	Oxidative stress, inflammation	5 mg/kg	20 mg/kg	4 weeks	4 weeks	Oral	IG	↑ kidney and body weight; SOD, CAT, GPx, GR, GSH, Nrf-2, HO-1, and γ-GCLC expression /activity ↓ blood urea nitrogen and serum creatinine; glomerulus shrinkage, tubule dilation, collagen deposition, and renal inflammation; COX-2, iNOS, PGE2, NO, EMT markers (TGF-β1, Twist, fibronectin)
Fu et al. (2017) [120] *	Sirt3/FoxO3a pathway	2 mg/kg	10 mg/kg	7 days	7 days	IP	IP	↑ mitochondrial biogenesis, membrane potential, mtDNA copy number and mass; ATP levels, COX IV, Sirt3, FoxO3a, PGC-1α, and SOD2 expression/activity ↓ mROS, Cd-induced apoptosis, and mitochondrial damage, caspase-3 activity, Bax expression, and ERK1/2 phosphorylation
Zhang et al. (2020) [122] *	Nuclear xenobiotic receptor (NXR) response and mitophagy	140 mg/kg	400 mg/kg	90 days	90 days	Oral	Oral	↑ CYP450 content, APND and ERND activity; activated NXRs response; mitochondrial function and structure; Sirt3, FoxO3a, PGC-1α, and SOD2 expression; T-SOD, Cu-Zn SOD, GSH-Px, GST, and CAT activities ↓ atrophy, enlargement, exfoliation, vacuolation, and nuclear damage; AH and NCR activities; oxidative stress markers; caspase-3 activity, Bax expression, ERK1/2 phosphorylation
Chou et al. (2019) [125] ^§^	Pyroptosis, ER stress	2–10 μM	10 μM	48 h	12 h	NA	NA	↑ SIRT1 protein levels and activity ↓ NLRP3, cleaved-caspase-1, cleaved-IL-1β; IL-6, IL-18, IL-1β, TNF-α; LDH release, PI-positive cells; ER stress markers; XBP-1s mRNA and protein levels, Edem1, P58ipk
Rafati et al. (2015) [131] *	[histological study]	1 mg/kg	20 mg/kg	Daily	4 weeks	IP	IP	↑ restoration of total hepatocyte volume and number, sinusoid and central vein volume. Restoration of total glomeruli volume, mean glomerulus volume, PCT and DCT volumes, and lengths. Restoration of glomerular changes and intact DCT length ↓ hepatocyte nuclei number, fibrous tissue accumulation and bridges, perisinusoidal fibrosis, canal narrowing. Degenerated glomeruli and tubules, fibrous tissue volume. Degenerative glomerular changes and dilatation of PCT and DCT lumen, fibrous tissue accumulation
Eybl et al. (2006) [132] *	Lipid peroxidation, GSH	7 mg/kg	20 mg/kg	Single	3 days	SC	Oral	↓ liver lipid peroxidation; glutathione levels and GPx activity; catalase activity
Al-Baqami et al. (2021) [133] *	Biomarkers of hepatic functions and antioxidant enzymes	5 mg/kg	20 mg/kg	30 days	30 days	IP	IP	↑ SOD, GPx, CAT ↑ALT, AST, LDH, ALP, γ-GT

*: in vivo; ^§^: in vitro; NA: not applicable; IP: intraperitoneal; IG: intragastric; SC: subcutaneous; ↑: increase; ↓: decrease/inhibition.

**Table 4:** Comprehensive summary of experimental designs employed in the articles reviewed in the thyroid section.

Article	Experimental Target System	Dosage		Exposure Time		Administration Route		Ameliorative Effects of Resveratrol
		Cd	RV	Cd	RV	Cd	RV	
Benvenga et al. (2020) [141] *	MCP-1; CXCL10	2 mg/kg	20 mg/kg	Daily	14 days	IP	Oral	Follicular area and epithelial height restored to control levels ↓ MCP-1/CCL2 and CXCL10 levels; perifollicular connective tissue
Benvenga et al. (2021) [142] *	[histological study]	2 mg/kg	20 mg/kg	Daily	14 days	IP	Oral	↑ follicular thyroid diameters ↓ number of CT-positive cells; TUNEL-positive C cells

*: in vivo; IP: intraperitoneal; ↑: increase; ↓: decrease/inhibition.

**Table 5:** Comprehensive summary of experimental designs employed in the articles reviewed in the multiorgan impact section.

Article	Experimental Target System	Dosage		Exposure Time		Administration Route		Ameliorative Effects of Resveratrol
		Cd	RV	Cd	RV	Cd	RV	
Mei et al. (2021) [150] ^§^	ERK1/2 and JNK pathways	10 μM	10 μM	48 h	48 h	NA	NA	↑ cell viability; differentiation; ALP, RUNX2, COL1, and BMP-2 activity/expression; p-ERK1/2 and p-JNK ↓ apoptosis and necrosis; phosphorylation of ERK1/2 and JNK
Sasikumar et al. (2023) [153] ^§^	Oxidative stress, MMP, Nrf2, and downstream genes	3 μM	20 μM	24 h	24 h	NA	NA	↑ cell viability; Nrf2 and downstream genes (HO-1, NQO1, SOD, CAT) ↓ Cd-induced structural abnormalities; expression of hypertrophic markers (ANP, BNP, βMHC)
Qian et al. (2021) [159] ^§^	EMT (in colorectal cancer)	1–10 μM	10–200 μM	24 h	24 h	NA	NA	↑ E-cadherin ↓ invasive ability of CRC cells; ZEB1, vimentin, and N-cadherin levels

^§^: in vitro; NA: not applicable; h: hours; MMP: mitochondrial membrane potential; EMT: epithelial–mesenchymal transition; ↑: increase; ↓: decrease/inhibition.
